# Implementation of Gate-All-Around Gate-Engineered Charge Plasma Nanowire FET-Based Common Source Amplifier

**DOI:** 10.3390/mi14071357

**Published:** 2023-06-30

**Authors:** Sarabdeep Singh, Leo Raj Solay, Sunny Anand, Naveen Kumar, Ravi Ranjan, Amandeep Singh

**Affiliations:** 1 Model Institute of Engineering and Technology, Jammu 181122, India; sarabdeep1611@gmail.com; 2 Department of Electronics and Communication Engineering, Amity University, Noida 201313, India; solayleoraj@gmail.com (L.R.S.); sunnyanand.42@gmail.com (S.A.); 3 Department of Electronics and Nanoscale Engineering, University of Glasgow, Glasgow G12 8QQ, UK; 4 Dr. B. R. Ambedkar National Institute of Technology, Jalandhar 144008, India; ranjan.ravi80@gmail.com; 5 National Institute of Technology, Srinagar 190006, India; amansingh@nitsri.ac.in

**Keywords:** charge plasma, single-material gate, dual-material channel, gate stacking, common source amplifier, Nanowire FET

## Abstract

This paper examines the performance of a Gate-Engineered Gate-All-Around Charge Plasma Nanowire Field Effect Transistor (GAA-DMG-GS-CP NW-FET) and the implementation of a common source (CS) amplifier circuit. The proposed GAA-DMG-GS-CP NW-FET incorporates dual-material gate (DMG) and gate stack (GS) as gate engineering techniques and its analog/RF performance parameters are compared to those of the Gate-All-Around Single-Material Gate Charge Plasma Nanowire Field Effect Transistor (GAA-SMG-CP NW-FET) device. Both Gate-All-Around (GAA) devices are designed using the Silvaco TCAD tool. GAA structures have demonstrated good gate control because the gate holds the channel, which is an inherent advantage for both devices discussed herein. The charge plasma dopingless technique is used, in which the source and drain regions are formed using metal contacts and necessary work functions rather than doping. This dopingless technique eliminates the need for doping, reducing fluctuations caused by random dopants and lowering the device’s thermal budget. Gate engineering techniques such as DMG and GS significantly improved the current characteristics which played a crucial role in obtaining maximum gain for circuit designs. The lookup table (LUT) approach is used in the implementation of the CS amplifier circuit with the proposed device. The transient response of the circuit is analyzed with both the device structures where the gain achieved for the CS amplifier circuit using the proposed GAA-DMG-GS-CP NW-FET is 15.06 dB. The superior performance showcased by the proposed GAA-DMG-GS-CP NW-FET device with analog, RF and circuit analysis proves its strong candidature for future nanoscale and low-power applications.

## 1. Introduction

With the rapid demand for increasing transistor counts upon integrated circuits (ICs), the scaling of the transistors has become crucial. With the decrease in the size of a transistor, we can accommodate a greater number of transistors, which facilitates a significant number of digital applications. Moore’s law, which was envisioned by Gordon Moore in 1965, states that, for every two and half years, the transistors count in an IC becomes exponentially doubled, with the cost being a constant factor. By increasing the scaling factor in Silicon-based Metal Oxide Semiconductor Field Effect Transistor (MOSFET) devices, short channel effects (SCEs) such as leakage current, lower current ratio and decrease in on-state current raised a few serious concerns. Researchers and scientists have found a significant number of alternative devices which can withstand the SCEs faced after scaling to the maximum number. A great deal of attention has been achieved by Gate-All-Around Silicon Nanowire Metal Oxide Semiconductor Field Effect Transistors (GAA Si NW MOSFETs) for their outstanding capability of downscaling the devices [1,2,3]. The Gate-All-Around (GAA) structures immensely dominated the devices such as Ultrathin body Single Gate MOSFETs (UTB SG MOSFETs), Dual-Gate or Double-Gate FETs (DG FETs) and Fin-shaped FETs (FinFETs) which were solutions for short channel effects (SCEs) [4,5] and poor gate control while scaling the technology [6,7,8]. Unlike the previously mentioned devices, the GAA device structures provide excellent electrostatic control, increased packaging density and resistance to SCEs, shedding a scope for device scaling at the nanometer scale [9,10]. The researchers began investigating GAA nanowire FET devices from a fabrication perspective wherein high series resistance from the formation of sharp junctions between highly doped source/drain and partially doped channel regions raised serious concerns [11,12].

Dopingless and junctionless devices offer promising prospects in the field of semiconductor technology and offer potential advantages over traditional devices. These new devices eliminate the need for doping by leveraging electrostatic gating and electric field modulation [9]. By carefully considering some key parameters such as Debye length, device thickness and metal work functions, these devices can exhibit excellent performance and open new possibilities for future electronic applications. Debye length is important for the operation of dopingless and junctionless devices. It represents the extent to which the electric field in a semiconductor material is effectively shielded. To achieve the effective control and modulation of the carriers, a Debye length comparable to or less than the thickness of the device is required. This ensures that the gate voltage electric field can affect the entire channel region effectively. Therefore, selecting the right semiconductor material with the desired internal carrier concentration and dielectric properties is critical to achieving the desired Debye length for optimal device performance [11]. The reduced thickness of the device reduces the distance between the gate electrode and the channel, allowing for stronger coupling between the gate voltage and the carrier concentration. However, there is a trade-off between device thickness and channel resistance, as a thinner channel can result in higher resistance and higher power dissipation. Therefore, careful optimization is required to find a balance between electrostatic control and overall device performance. Choosing a metal with a work function that is well-matched to the energy levels of the semiconductor material is critical to minimizing energy barriers. This balancing ensures the efficient transport and modulation of the carrier, thereby increasing the efficiency of the device. In addition, the right metal gate design can alleviate issues such as Fermi-level pinning and contact resistance, further improving the overall characteristics of the device [13,14].

With extensive research in device modelling, junctionless nanowire FETs (JL NW FETs) without physical junctions were introduced as a solution to the high series problem in GAA devices. However, the need for doping to form source, channel and drain regions in JL NW FETs resulted in a reduction in carrier mobility due to the fluctuations caused by random dopants (RDFs) [13]. Heavy doping has become a serious issue as it degrades the device’s performance. The issue raised by JL NW FETs can be addressed by a dopingless technique called charge plasma [14,15]. This dopingless concept has been applied to MOSFETs, Tunnel FETs (TFETs) and to GAA NW FETs [16]. The charge plasma dopingless technique eliminates the need for doping to create source and drain regions. Instead of doping, metals were placed and assigned a suitable work function to serve as source and drain metals. This technique not only solves the doping control issues, but also provides a low thermal budget, reduced SCEs and improved current characteristics [16]. By controlling the RDFs, charge plasma-based devices showcased better electrical characteristics than junctionless devices.

Gate engineering techniques are predominantly used to improve device characteristics. The rapid acceleration in the charge carriers in the channel can be increased by adding another gate to the existing gate, making it a dual-material gate (DMG) [17]. These two laterally contacting metals with different work functions give a boost to the drain current as the electric field along the channel becomes more uniform and the electrons near the source are accelerated more rapidly. As a result, the average electron velocity in the channel is increased. DMG structures, when compared to the single-material gate (SMG), offer better transconductance and reduced SCEs. The scaling factor of the device is also hindered by a gate dielectric, silicon dioxide of less than 2 nm. This limit is due to quantum mechanical tunnelling. This scaling issue can be resolved by adding a high-k material upon the gate dielectric later, which is commonly known as the gate stack (GS) technique [18,19]. A significant number of studies have reported higher analog performance of using DMG and GS collectively. Thus, combining the charge plasma dopingless, DMG and GS techniques with GAA-SMG-CP NW-FET gives rise to a new device configuration, namely Gate-All-Around Dual Material Gate–Gate stack Charge Plasma Nanowire FET (GAA-DMG-GS-CP NW-FET).

The main aim of this paper is to propose and discuss the design aspects of the GAA-DMG-GS-CP NW-FET and to implement and investigate a common source (CS) amplifier circuit designed using GAA-DMG-GS-CP NW-FET and compared with a GAA-SMG-CP NW-FET device. Field effect transistors (FETs) are a good choice for designing digital ICs. Enormous groups in the past have worked on implementing SRAM circuits. In this work, the potential of the devices for implementation of CS amplifier circuits and their analysis for designing analog integrated circuits are investigated. Initially, the analog and RF analysis of the GAA-DMG-GS-CP NW-FET is carried out to analyze the effect caused by adding these gate-engineering techniques to the GAA CP NW FET device. Later, a CS amplifier circuit is implemented using the lookup table (LUT) approach using Verilog-A on both devices. The LUT-based Verilog model has been utilized by numerous research groups for carrying out circuit implementations. The LUT-based Verilog model used the transfer and capacitance characteristics of the device that are to be implemented [19,20]. The effect of added gate-engineering techniques in the circuits is discussed and the gain of the proposed GAA-DMG-GS-CP NW-FET is compared to its counterpart.

The rest of this paper is structured as follows: the proposed GAA-DMG-GS-CP NW-FET device’s specifications and simulation parameters are discussed in Section 2. In Section 3, the results and discussion in accordance with the enhancement caused by adding gate-engineering techniques to the GAA-SMG-CP NW-FET device followed by the LUT approach for implementing the circuit and its analysis are presented. A fair conclusion is drawn from the results provided in the Section 4.

## 2. Device Structure and Simulation Parameters

**Figure 1:**
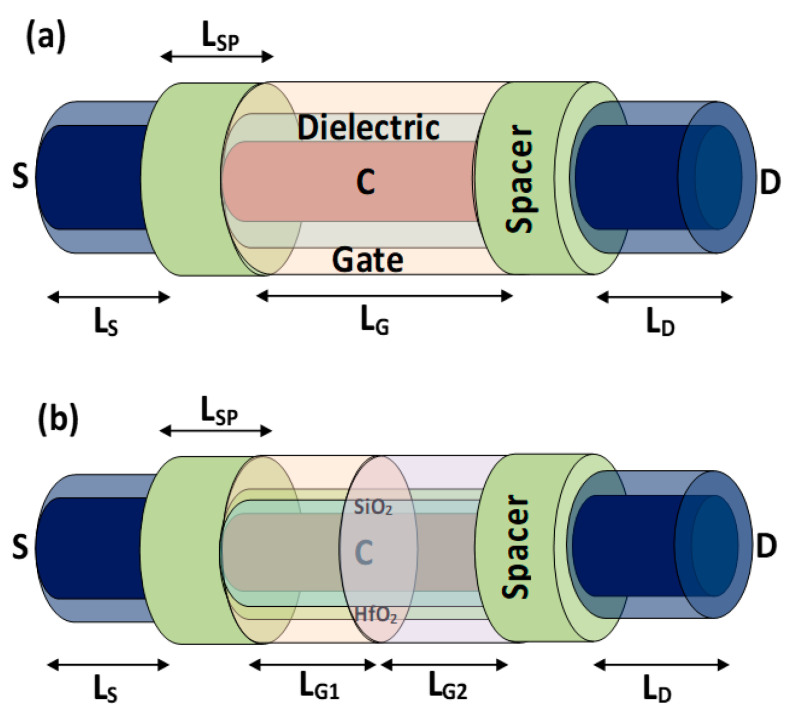
Three-dimensional schematics of (**a**) GAA-SMG-CP NW-FET and (**b**) GAA-DMG-GS-CP NW-FET.

**Figure 2:**
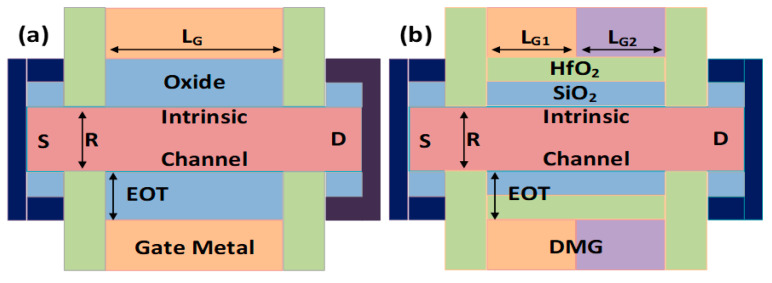
Cross-sectional view of (**a**) GAA-SMG-CP NW-FET and (**b**) GAA-DMG-GS-CP NW-FET.

The three-dimensional schematics of GAA-SMG-CP NW-FET and GAA-DMG-GS-CP NW-FET are shown in Figure 1a,b, respectively. A cross-sectional view of device structures [21] is shown in Figure 2 as (a) GAA-SMG-CP NW-FET and (b) GAA-DMG-GS-CP NW-FET. Both the devices have the same silicon body thickness (t_Si_) of 10 nm, which is usually intrinsic in nature, the gate length (L_G_) of GAA-SMG-CP NW-FET is 20 nm as well as for the GAA-DMG-GS-CP NW-FET that uses DMG technique [22,23]; hence, the gate length L_G1_ and L_G2_ are considered as 10 nm each. The gate oxide thickness (t_OX_) is of 1 nm for GAA-SMG-CP NW-FET as well as for the GAA-DMG-GS-CP NW-FET which uses the gate stack technique; thus, the t_OX_ + t_high-K_ takes the advantage of the equivalent oxide thickness (EOT) of 1 nm. Silicon dioxide is the gate dielectric and hafnium oxide is the high-K material used for the implementation of the gate stack technique. Apart from the addition of gate-engineering techniques, the remaining parameters are taken equally for both devices.

The gate metal work function (ϕ_G_) of GAA-SMG-CP NW-FET is taken as 4.63 eV (Niobium) as well as for the GAA-DMG-GS-CP NW-FET which has dual gates, while the work function of gate 1 (ϕ_G1_) and (ϕ_G2_) is 4.66 (Silver) and 4.16 (Tantalum), respectively. For a fair comparison, the work functions of the gate metals are set to have the same threshold voltage (V_T_) value at a drain voltage (V_D_) of 0.5 V. For the best possible outcomes, the work function differences of both the metals of GAA-DMG-GS-CP NW-FET are kept at 0.5 eV. The channel length (L_C_) is of 20 nm and, for both devices, GAA-SMG-CP NW-FET and GAA-DMG-GS-CP NW-FET being nanowire structures, a radius of 5 nm is taken into consideration. The remaining parameters for both devices are collectively shown in Table 1.

**Figure 3:**
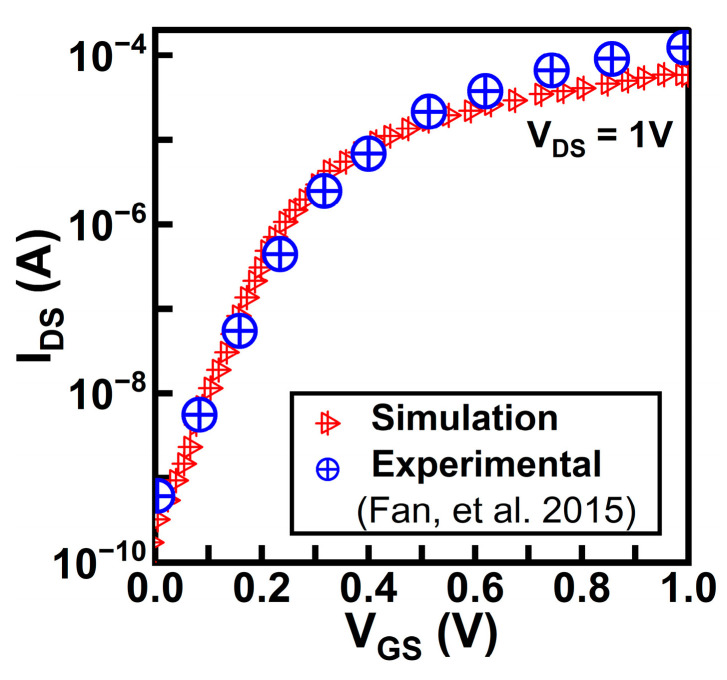
Transfer characteristics in log scale simulation and experimental data reported in [24] of nanowire FETs.

For creating charge plasma-based device structures, the undoped intrinsic silicon body is induced with N+ source and drain regions. There are certain conditions to be followed for the generation of electron charge plasma, namely the work function of silicon (ϕ_Si_) should be greater than the work functions of the source and drain regions and the substrate body length should be less than that of Debye length. To satisfy these conditions, one such possibility is that the work function of the source (ϕ_S_) and drain (ϕ_D_) regions be set to hafnium 3.9 eV and the silicon thickness be set as 10 nm for creating charge plasma-based nanowire FET devices. Both devices, GAA-SMG-CP NW-FET and GAA-DMG-GS-CP NW-FET, have considered the same limitations, such as the use of the same work functions and thickness of the silicon body. Hafnium dioxide (HfO_2_) is considered a high-K material under the metal electrodes of the source and drain spacers. The simulation transfer curve calibrated with the experimental data [24] of nanowire FETs is shown in Figure 3. A clear idea in [25] gives the step-by-step process of the silicon nanowires’ fabrication.

Simulations were carried out using the Silvaco Atlas TCAD device simulator [21]. Auger and SRH models are utilized for the minority charge carrier recombination model. Concentration-dependent mobility and high-field reduction mobility were used for mobility models. The Bohm quantum model is considered for quantum confinement effects.

## 3. Results and Discussion

**Figure 4:**
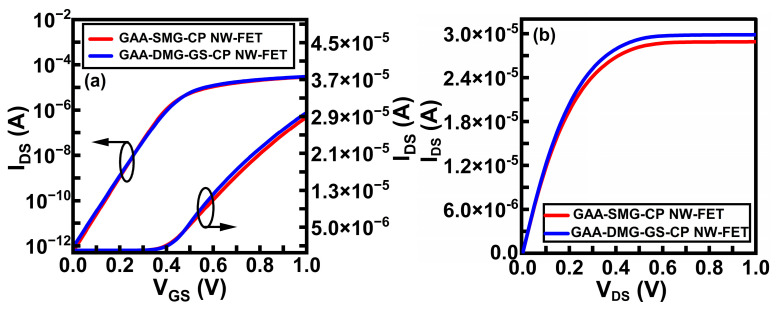
(**a**) I_DS_ versus V_GS_ characteristics and (**b**) I_DS_ versus V_DS_ (linear) output characteristics of GAA-SMG-CP NW-FET and GAA-DMG-GS-CP NW-FET.

As Figure 4a illustrates a graph between the drain current (I_DS_) and the gate voltage (V_GS_) of the GAA-DMG-GS-CPNW-FET and the GAA-SMG-CP NW-FET devices at V_DS_ = 1 V. The GAA-DMG-GS-CP NW-FET device shows a higher ON-state current of 3 × 10^−5^ A/µm due to the addition of the DMG and GS techniques. Since the GS technique is employed in the GAA-DMG-GS-CP NW-FET device, it shows a low off-current compared to the GAA-SMG-CP NW-FET device.

Figure 4b shows the comparative linear output (I_DS_ versus V_DS_) characteristics plots of the GAA-SMG-CP NW-FET and the GAA-DMG-GS-CP NW-FET devices. The first curve corresponds to the ON-state current of GAA-DMG-GS-CP NW-FET at V_GS_ = 1 V, which is higher than GAA-SMG-CP NW-FET due to gate stacking and the dual-gate dielectric material on the gate side with a high band gap of 25 eV.

The use of a dual metal gate and gate stacking in a nanowire FET (field-effect transistor) can slightly decrease the subthreshold slope, which is a measure of how rapidly the device switches from the OFF-state to the ON-state. With dual metal gates and gate-stacking, it is possible to optimize surface potential to precisely control the threshold voltage and enhance the gate control efficiency. However, even with better channel control, the dual metal gate can affect the source and drain region via fringing fields across the gate stack and spacers. The gate–source coupling increases for the combination of gate-work functions which degraded the switching characteristics by a minimal value providing better ON-current as a trade-off [16].

Another significant parameter is transfer conductance or transconductance [16]. It is the electrical characteristic that is reciprocal to the trans-resistance, denoted by g_m_, which relates the drain current to the change in input voltage at a constant drain voltage of 1 V.

g_m_ = (∂I_D_)/(∂V_GS_)|at V_DS_, constant (1)

**Figure 5:**
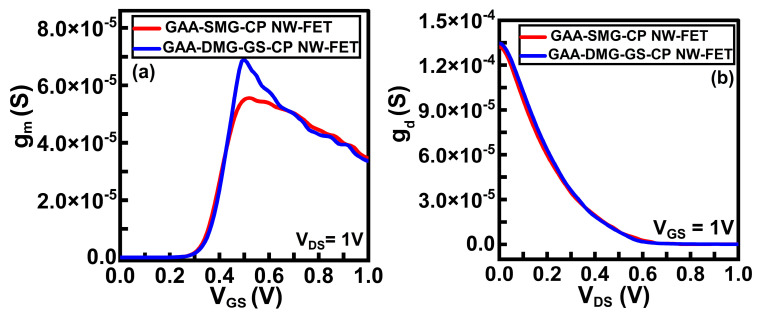
(**a**) Transconductance and (**b**) output conductance of GAA-SMG-CP NW-FET and GAA-DMG-GS-CP NW-FET.

Figure 5a shows the transconductance of the GAA-DMG-GS-CP NW-FET device in comparison to that of the GAA-SMG-CP NW-FET device. It is observed from the plot that g_m_ is higher for GAA-DMG-GS-CP NW-FET. High transconductance means the fastest transistor in terms of speed, which further makes the device a good choice for high-frequency applications. It can also be noticed from the plot that the transconductance attains its maximum value at a gate voltage of 0.5 V.

The output conductance [16], g_d_, represents the change in output current caused by a small change in drain voltage with a constant gate voltage of 1 V.

g_d_ = (∂I_DS_)/(∂V_DS_)|at V_GS_, constant (2)

**Figure 6:**
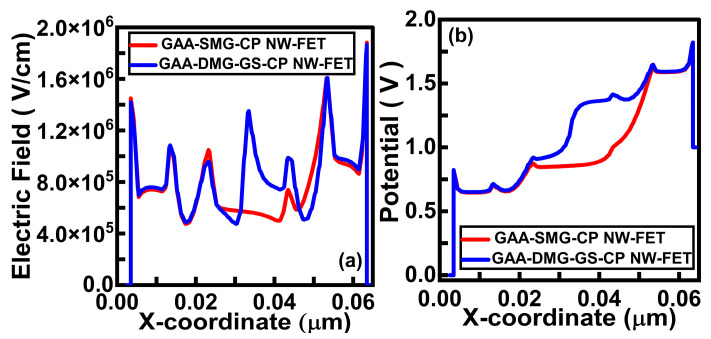
(**a**) Electric field and (**b**) potential of GAA-SMG-CP NW-FET and GAA-DMG-GS-CP NW-FET.

Output conductance is also called drain conductance. Figure 5b shows the output conductance plots of the GAA-SMG-CP NW-FET device and the GAA-DMG-GS-CP NW-FET device. GAA-DMG-GS-CP NW-FET delivers comparatively higher output conductance gain due to the high electron carrier mobility and output current characteristics. Figure 6a shows the comparative electric field plots of the GAA-SMG-CP NW-FET and GAA-DMG-GS-CP NW-FET devices at V_GS_ = 1 V and V_DS_ = 1 V. GAA-DMG-GS-CP NW-FET shows a higher value of the electric field at the channel region, which indicates a higher drain current.

The potential of GAA-DMG-GS-CP NW-FET shows enhanced values compared to GAA-SMG-CP NW-FET in Figure 6b, due to the use of the DMG technique, which indicates more passage of electrons through a potential barrier from the source to channel region. A stronger electric field indicates a high flow of drain current.

**Figure 7:**
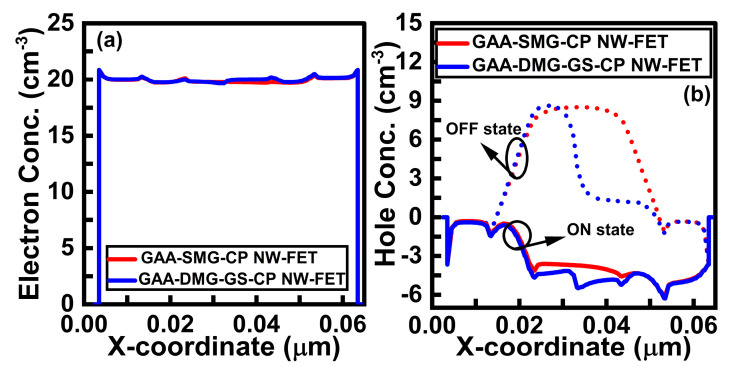
(**a**) Electron concentration (log scale) and (**b**) hole concentration (log scale) of GAA-SMG-CP NW-FET and GAA-DMG-GS-CP NW-FET devices at onset state (V_GS_ = V_DS_ = 1 V) and offset state (V_DS_ = 1 V, V_GS_ = 0 V).

Figure 7a shows the plot of electron concentration at V_GS_ = V_DS_ = 1 V. The GAA-DMG-GS-CP NW-FET device shows a higher electron concentration due to the high electron carrier mobility characteristics. Figure 7b shows the plot of hole concentration; the dotted line and solid line indicate the plot at the offset and onset state, respectively.

The GAA-DMG-GS-CP NW-FET device has the lowest minority charge carriers in both the source region and drain region at the offset state. The hole concentration is highest for GAA-SMG-CP NW-FET in both offset and onset states, as shown in Figure 7b.

**Figure 8:**
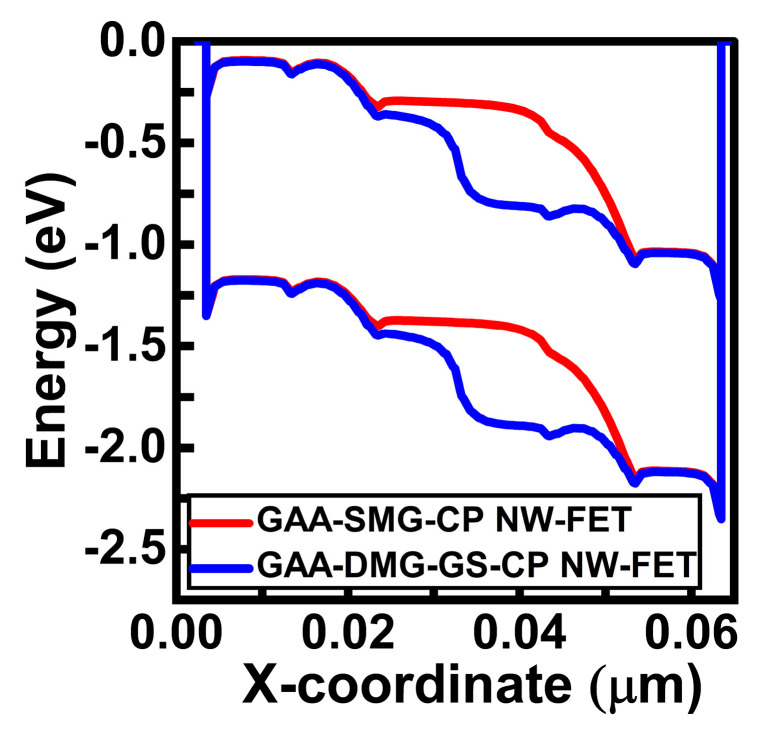
The energy of GAA-SMG-CP NW-FET and GAA-DMG-GS-CP NW-FET devices at V_GS_ = 1 V.

Figure 8 shows the comparative energy diagrams of the GAA-SMG-CP NW-FET and the GAA-DMG-GS-CP NW-FET devices at the onset condition. When gate voltage crosses the threshold voltage value, these structures are in an onset state. This will lead to the flow of electrons across the junction and the rising of the current. The proposed device has shown higher current due to the alignment of both the valence band and conduction band of the intrinsic region to the drain region. The electrostatic behavior of the GAA-DMG-GS-CP NW-FET device with the gate-engineering techniques of DMG and GS regarding RF and analog parameters is discussed below. The sum of $C_{gs}$ and $C_{gd}$ capacitances indicate total gate capacitance. Thus, the total gate capacitance, $C_{gg}$ is depicted as [16]

(3) $$C_{gg}=C_{gs}+C_{gd}$$

**Figure 9:**
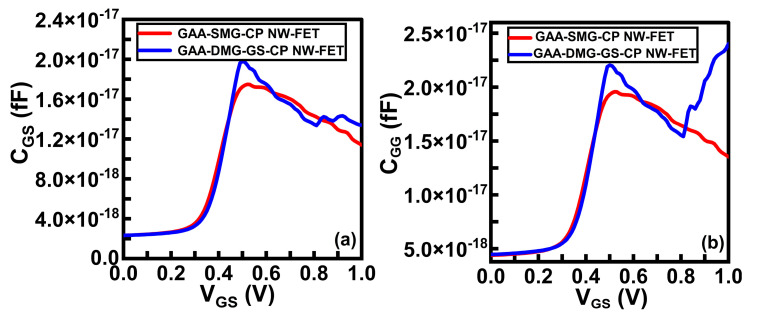
(**a**) Gate-to-source capacitance and (**b**) total gate capacitance of GAA-SMG-CP NW-FET and GAA-DMG-GS-CP NW-FET.

These parasitic or intrinsic capacitances affect the switching performance of the device. Figure 9a,b show the plots of the capacitance from gate to source (C_gs_) and total gate capacitance (C_gg_), respectively, with respect to V_GS_. It is clearly seen from both plots that GAA-DMG-GS-CP NW-FET possesses a maximum gate-to-source capacitance of 2 × 10^−17^ and a total gate capacitance of 2.3 × 10^−17^ at a V_GS_ of 0.5 V.

The computer-aided design tool, Smart Spice, has been used to design the lookup table (LUT) based Verilog-A model to carry out spice simulation of the common source circuit [18]. A lookup table is a customized table that uses the V_DS_, V_GS_, I_DS_, and values of the FET device to generate this table in LUT form [20]. A total of three lookup tables are required, which can be extracted from the device simulations for I_DS_, C_GS_ and C_GD_. These lookup tables are utilized in the Verilog-A code, which is in turn used in the spice code for carrying out the analysis of the FET-based common source amplifier circuit. Finally, for generating the output of the common source amplifier FET circuit, this spice file is loaded into Smart Spice, and the output waveform is in the Smart View [22,23].

**Figure 10:**
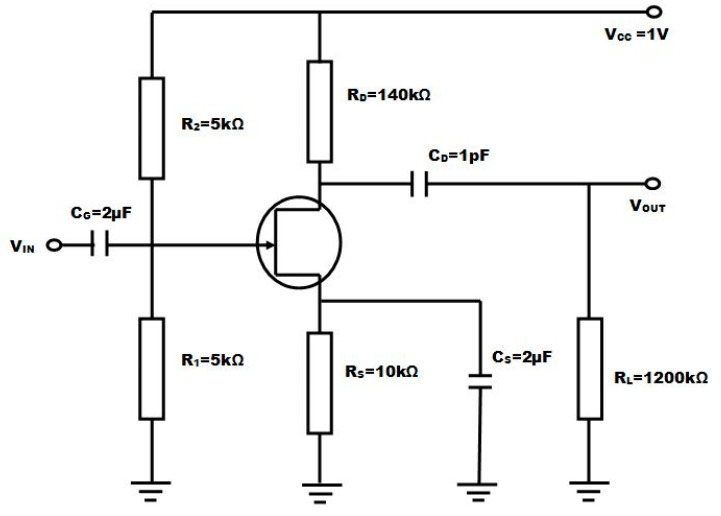
Common source (CS) amplifier circuit diagram.

An amplifier is a device used to increase the strength of a weak signal. Amplification is an essential function of an analog circuit. Figure 10 depicts the common-source amplifier circuit diagram with resistive load, which is designed using GAA-DMG-GS-CP NW-FET. A CS amplifier circuit is designed using a load resistance R_L_ of 1200 kΩ, a drain resistance R_D_ = 140 kΩ and a source resistance R_S_ = 10 kΩ with a drain capacitance C_D_ of 1 pF, which is used to remove the DC component from the output signal. The sine-wave input voltage applied at the gate terminal of the common source amplifier circuit is kept at 50 mV with a frequency of 1 MHZ.

The gain for the common source (CS) amplifier circuit is expressed as

A_V_ = −g_m_ R_L_′, R_L_′= R_D_||R_L_

where R_L_′ represents the parallel combination of the drain resistance and load resistance. From the above gain equation, it is concluded that the gain of a CS amplifier is dependent on the equivalent load resistance (R_L_′) and transconductance of the device. Here, the analysis and comparison of the transient response of the GAA-SMG-GS-CP NW-FET and GAA-DMG-GS-CP NW-FET CS amplifier circuits have been carried out. Furthermore, the transient analysis comparison of the proposed CS amplifier circuit is performed with that of amplifiers based on GAA-DMG-GS-CP NW-FET, MOSFET and Hetero dielectric-tri material gate tunnel FET (HD-TMGTFET) by keeping a load resistance at 1000 KΩ. Comparisons will be carried out in terms of various important parameters such as gain, amplification factor, etc. An amplification factor is defined as the ratio of the output voltage to the input voltage. An amplification factor is known as gain when it is expressed in logarithmic dB units. These parameters can be written as [20]

(4) $$\text{Amplification factor}=\frac{V_{\text{out}}}{V_{\text{in}}}$$

(5) $$Gain(dB)=20{log}_{10}\frac{V_{out}}{V_{in}}$$

**Figure 11:**
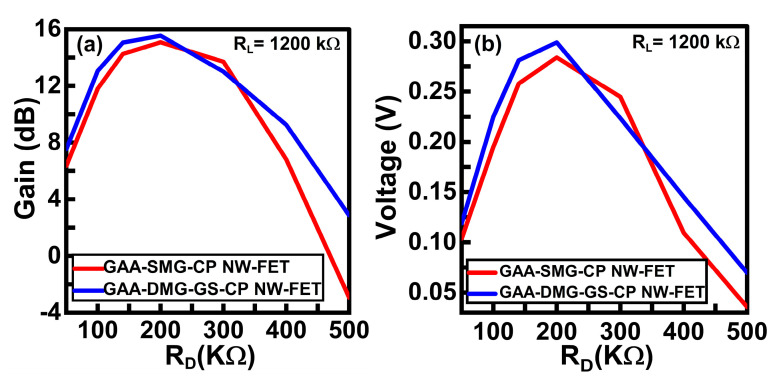
(**a**) Voltage versus R_D_ plot and (**b**) gain versus R_D_ plot at constant R_L_ value of 1200 kΩ for GAA-SMG-CP NW-FET and GAA-DMG-GS-CP NW-FET CS circuits.

Figure 11a,b represent the variation in voltage and gain with respect to drain resistance, respectively, at a constant load value of 1200 kΩ. From both graphs, with an R_D_ of 140 kΩ, the proposed CS amplifier circuit shows a higher voltage and gain value than the GAA-DMG-GS-CP NW-FET CS amplifier circuit. With this analysis, the R_D_ value of 140 kΩ is chosen as the appropriate value for designing the proposed CS amplifier circuit.

**Figure 12:**
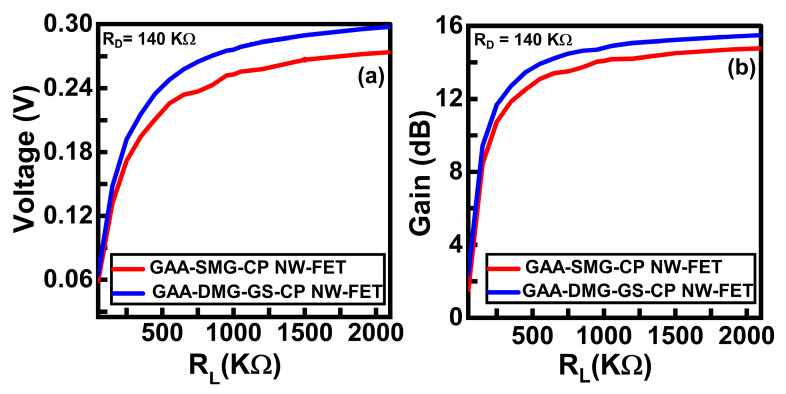
(**a**) Voltage versus R_L_ plot and (**b**) gain versus R_L_ plot at constant R_D_ value of 140 kΩ for GAA-SMG-CP NW-FET and GAA-DMG-GS-CP NW-FET CS circuits.

The graphs of voltage with respect to R_L_ and gain with respect to R_L_ at a constant drain value of 140 kΩ are displayed in Figure 12a,b, respectively. Here, extensive analysis is conducted for selecting the appropriate values of R_D_ and R_L_**.** The load resistance value is taken as 1200 kΩ for carrying out the transient analysis of GAA-SMG-CP NW-FET and GAA-DMG-GS-CP NW-FET CS amplifier circuits.

**Figure 13:**
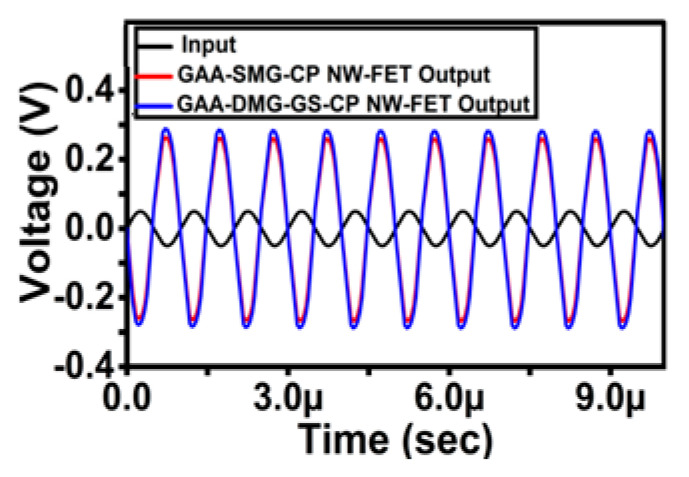
Transient analysis of GAA-DMG-GS-CP NW-FET with GAA-SMG-CP NW-FET common-source circuit at R_L_ = 1200 kΩ.

Figure 13 shows the transient analysis comparison plot of the GAA-SMG-CP NW-FET and GAA-DMG-GS-CP NW-FET amplifiers at R_D_ = 140 kΩ and R_L_ = 1200 kΩ. The plot shows that the proposed device-based CS amplifier circuit exhibits a high amplification factor of up to 5.6 times within the frequency range from 1 MHz to 10 MHz. Similarly, the GAA-SMG-CP NW-FET-based CS amplifier attains a lesser amplification factor of 5.1 times as compared to the GAA-DMG-GS-CP NW-FET-based CS amplifier.

Table 2 compares the performance of the proposed GAA-DMG-GS-CP NW-FET-based common-source amplifier circuit to that of the SMG-CP NW-FET CS circuit. The proposed GAA-DMG-GS-CP NW-FET common-source amplifier circuit provides a high amplification of 15.06 dB in terms of gain, whereas the GAA-SMG-CP NW-FET-based CS amplifier delivers 14.25 dB of gain due to the high on-drive current. The proposed GAA-DMG-GS-CP NW-FET and SMG-CP NW-FET exhibited excellent amplification factors. The gain has been increased with the help of gate-engineering techniques such as dual-material gate and gate stack added to the proposed device. Additionally, the charge plasma dopingless aided in reducing the random dopant fluctuations and thermal budgeting.

**Figure 14:**
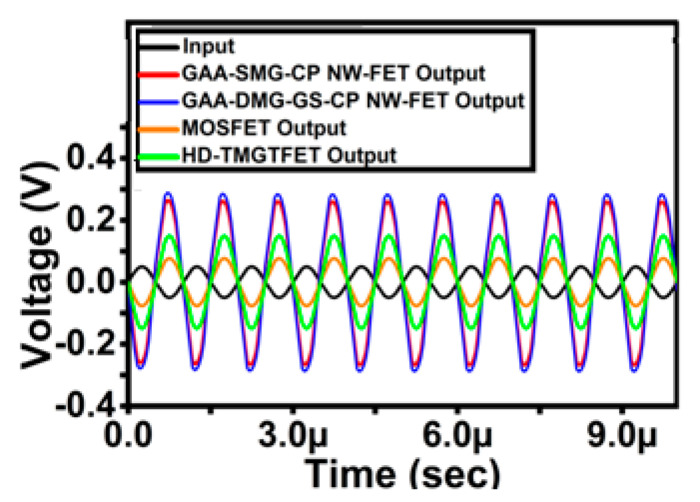
Transient analysis of GAA-DMG-GS-CP NW-FET with GAA-SMG-CP NW-FET, MOSFET [23] and HD-TMG TFET [23] common-source circuits at R_L_ = 1000 kΩ.

Figure 14 shows the transient analysis comparison of the proposed CS amplifier circuit with that of amplifiers based on GAA-SMG-GS-CP NW-FET, MOSFET [23] and HD-TMGTFET [23] CS circuits. Here, the load resistance value is kept at 1000 kΩ. The transient analysis comparison result shows that the proposed CS amplifier-based circuit exhibits a high amplification factor of up to 5.02 times within the frequency range from 1 MHz to 10 MHz. The gain of the GAA-SMG-CP NW-FET, MOSFET [23] and HD-TMGTFET [23]-based CS amplifier circuits is 13.40 dB, 9.18 dB and 3.40 dB, respectively, while the proposed circuit has shown a better gain of 14.01 dB. Table 3 shows the performance comparison of the proposed common-source amplifier circuit in terms of output voltage, gain and amplification factor with GAA-SMG-GS-CP NW-FET, MOSFET [23] and HD-TMGTFET [23]-based CS circuits. The devices GAA-SMG-GS-CP NW-FET and the proposed GAA-DMG-GS-CP NW-FET, as gate-all-around structures, have good gate controllability of the channel, which in turn reduces the short-channel effects and gives the better on-current and current ratios, which is the prime reason for achieving better gain when compared to the other devices.

The comparative analysis with that of the proposed CS amplifier circuit GAA-DMG-GS-CP NW-FET gives a better outcome in terms of output voltage and gain when compared with GAA-SMG-CP NW-FET, MOSFET [23] and HD-TMGTFET [23]-based amplifier circuits. Hence, the GAA-DMG-GS-CP NW-FET device-based CS amplifier circuit can be used for designing amplifiers, which is one of the most crucial building blocks of analog ICs.

## 4. Conclusions

This paper proposes the design of a common-source (CS) amplifier circuit using gate-engineering techniques such as dual-material gate and gate stacking upon a gate-all-around nanowire FET structure, GAA-DMG-GS-CP NW-FET. These gate-engineering techniques improve the device characteristics, which aids in improving the circuit behavior. Gate-all-around structures are well known for their good gate controllability over the channel due to their gate-surrounding architectures. The charge plasma dopingless techniques not only reduce the random dopant fluctuations but also reduce the thermal budget. In comparison to the single-material gate, the GAA-SMG-CP NW-FET structure, the analysis of the transfer characteristics shows that the proposed GAA-DMG-GS-CP NW-FET device performs better in terms of ON-state current, at 3 × 10^−5^ A/µm, and of lowest Off-state current, at 10^−13^ A/µm. The LUT-based Verilog model is used for circuit implementation. The transient response of the CS amplifier circuit implemented using GAA-SMG-CP NW-FET and GAA-DMG-GS-CP NW-FET shows outstanding characteristics with gains of 14.25 dB and 15.03 dB, respectively, at R_D_ = 140 kΩ and R_L_ = 1200 kΩ. The proposed GAA-DMG-GS-CP NW-FET device-based common-source amplifier gain attribute is compared with other device-based CS amplifier circuits, which proved its promising candidature for designing future nanoscale high-amplification circuits.

## Figures and Tables

**Table 1:** Device specification and design parameters.

Device Parameters	GAA-SMG-CP NW-FET	GAA-DMG-GS-CP NW-FET	
Channel length (L_C_)	20 nm	20 nm	
Nanowire radius (R)	5 nm	5 nm	
Gate length (L_G_)	20 nm	L_G1_	L_G2_
		10 nm	10 nm
Gate metal work function (ϕ_G_)	4.63 eV	ϕ_G1_	ϕ_G2_
		4.66 eV	4.16 eV
Gate oxide thickness (t_OX_)	1 nm	t_OX1_	t_OX2_
		0.5 nm	0.5 nm
Gate oxide material	SiO_2_	SiO_2_	HfO_2_
Source/Drain length (L_S_/L_D_)	10 nm	10 nm	
Source/Drain work function	3.9 eV	3.9 eV	
Thickness of silicon (t_Si_)	10 nm	10 nm	
Doping	1 × 10^15^ cm^−3^	1 × 10^15^ cm^−3^	
Spacer length (L_SP_)	10 nm	10 nm	
Spacer material	Hafnium	Hafnium	

**Table 2:** Performance analysis of SMG CP GAA NWFET and DMG GS CP GAA NWFET-based CS amplifier circuits.

CS Amplifier Circuit	R_L_	V_OUT_	GAIN (dB)
SMG-CP-GAA NW-FET	1200 kΩ	0.258 V	14.25
GAA-DMG-GS-CP NW-FET	1200 kΩ	0.283 V	15.06

**Table 3:** Performance parameters for various CS amplifier circuits.

CS Amplifier Circuit	R_L_	V_OUT_	Amplification Factor	GAIN (dB)
GAA-DMG-GS-CP NW-FET	1000 kΩ	0.251 V	5.02 times	14.01 dB
GAA-SMG-CP NW-FET	1000 kΩ	0.234 V	4.68 times	13.40 dB
HD-TMGTFET [23]	1000 kΩ	0.144 V	2.89 times	9.18 dB
MOSFET [23]	1000 kΩ	0.074 V	1.48 times	3.40 dB

## Data Availability

Data will be provided upon reasonable request.
